# In Vitro Assessment of Corrosion Rate, Vickers Hardness and SEM Analysis of Glass Ionomer Cements and Calcium Silicate-Based Materials

**DOI:** 10.3390/bioengineering12111261

**Published:** 2025-11-18

**Authors:** Diana Hanu, Sorina Mihaela Solomon, Simona Stoleriu, Alice Murariu, Nicanor Cimpoeșu, Gianina Iovan

**Affiliations:** 1 Fixed Restorations, Department of Odontotherapy-Periodontology, Faculty of Dentistry, Grigore T. Popa University of Medicine and Pharmacy Iasi, 700115 Iasi, Romania; diana.roman@umfiasi.ro (D.H.); sorina.solomon@umfiasi.ro (S.M.S.); simona.stoleriu@umfiasi.ro (S.S.); gianina.iovan@umfiasi.ro (G.I.); 2 Department of Surgicals, Faculty of Dentistry, Grigore T. Popa University of Medicine and Pharmacy Iasi, 700115 Iasi, Romania; alice.murariu@umfiasi.ro; 3 Faculty of Material Science and Engineering, “Gheorghe Asachi” Technical University of Iași, 700050 Iasi, Romania

**Keywords:** calcium silicate, corrosion rate, glass ionomer, SEM analysis, Vickers hardness

## Abstract

The long-term stability of bioactive dental cements in acidic environments is not yet fully understood, despite their extensive clinical use in restorative and endodontic procedures. The objective of this study is to evaluate the degradation behaviour and mechanical stability of one glass ionomer cement (GC FUJI IX^®^) and two calcium-silicate-based materials (Biodentine^®^ and Biodentine XP 500^®^) under simulated acidic oral conditions. A total of 18 samples were prepared and distributed into three groups. The materials were immersed in a solution with a pH of 4.5, and their performance was assessed through a number of different methods. These included mass-loss measurements, corrosion-rate calculations, Vickers microhardness testing, and SEM to characterise the surfaces. Biodentine^®^ exhibited the highest degradation, followed by Bio-Dentine XP 500^®^ and GC FUJI IX^®^. The data were confirmed by one-way ANOVA and a post hoc Tukey’s test. This indicated a statistically significant superiority (*p* < 0.05) of Biodentine XP 500^®^ over glass ionomers in terms of surface hardness maintenance under acidic conditions. Biodentine^®^, a calcium silicate-based material, demonstrated inferior chemical stability compared to GC FUJI IX^®^ and Biodentine XP 500^®^, likely due to its modified calcium-silicate formulation that limits ionic dissolution. In addition, the study revealed that Biodentine XP 500^®^ exhibited the highest Vickers hardness under acidic conditions. The findings reported in this study offer valuable insights into the material selection process for low-pH clinical scenarios and contribute to a more comprehensive understanding of the chemical–mechanical stability of modern bioactive dental restoratives.

## 1. Introduction

In contemporary dentistry, a fundamental objective is the creation of restorative materials that guarantee an aesthetically pleasing outcome. It is imperative that these materials exhibit bioactive properties and possess sufficient structural, mechanical and chemical stability to endure the complex conditions of the oral environment. Glass ionomer- and calcium silicate-based materials are regarded as highly valuable due to their exceptional characteristics and their positive long-term contributions to oral health [1].

Glass ionomers, which were introduced into dental practice in 1970, remain a popular choice in the field of dental restorations due to their ability to chemically bond to dental structures and their capacity to release fluoride [2]. The release of fluoride is of pivotal significance in the prevention of carious lesions. This is achieved through the facilitation of the remineralisation process and the protection of enamel from cariogenic action [3].

Moreover, calcium silicate-based materials have witnessed a marked increase in popularity, largely attributable to their noteworthy biocompatibility and capacity to stimulate dentin regeneration [4]. These characteristics are of particular significance within the domain of direct and indirect pulp-capping [5]. Recently, novel formulations have been developed that have the potential to be utilised as dental restorative materials, thereby obviating the necessity for immediate coverage with a long-term restorative material. The physico-chemical properties of calcium silicate, in conjunction with its favourable impact on biological tissues, render it a material of significant value for restorative applications [6].

The chemical stability of these materials is a crucial characteristic that influences their durability and clinical performance [7]. Dental materials are frequently exposed to pH fluctuations, temperature changes and contact with a variety of substances in the oral cavity. These factors have the potential to compromise the integrity of the materials [8]. Recent advancements in the chemical structure of glass ionomers and calcium silicate have led to novel developments in dental materials, emphasising the potential for enhancing chemical stability, which, in turn, reflects their clinical efficacy. The modifications made to these materials have two principal consequences. Firstly, they improve their fundamental properties. Secondly, they contribute to their resistance to the challenges encountered in the oral environment [9].

The incorporation of nanometer-sized particles into the composition of dental materials represents a significant development in emerging technologies. This approach has been demonstrated to enhance the resistance of materials to solubility and abrasion. These are two critical factors in ensuring the maintenance of material integrity and functionality when in contact with oral fluids and under varying conditions of use [10]. In this context, the present in vitro study aims to analyse these materials under controlled conditions that replicate situations encountered in the oral environment. The objective is to understand the chemical and mechanical behaviour of glass ionomer- and calcium silicate-based restorative materials. 

The selection of Biodentine^®^, Biodentine XP 500^®^ and GC FUJI IX^®^ for this study was deliberate, reflecting their widespread use and distinct characteristics within the field of restorative dentistry. Biodentine^®^, a calcium silicate-based cement, has been demonstrated to exhibit both biocompatibility and bioactivity, rendering it an appropriate material for use in pulp capping [11] and dentin regeneration [12]. Biodentine XP 500^®^, a modified version incorporating advanced technology, claims enhanced physicochemical properties and handling [13]. GC FUJI IX^®^, a conventional glass ionomer cement, remains a popular choice due to its fluoride release [14] and chemical adhesion to tooth structure [2]. Whilst earlier studies have examined each material in isolation, a thorough investigation into the comparative corrosion resistance and hardness of these materials under simulated oral conditions remains to be conducted. The objective of this study is to address the identified knowledge gap by means of a direct comparison of the three materials under consideration. The study will offer valuable insights into their suitability for various clinical applications and will contribute to the selection of materials in restorative dentistry on the basis of evidence.

## 2. Materials and Methods

### 2.1. Materials

The materials employed in this study were procured from the commercial sector as follows: GC Fuji IX^®^ (GC Corporation^®^, Tokyo, Japan), Biodentine^®^ (Septodont^®^, Lancaster, PA, USA) and Biodentine XP 500^®^ (Septodont^®^, Lancaster, PA, USA). In order to guarantee that the samples possessed a standardised geometry, cylindrical polyethylene moulds measuring 10 mm in length × 4 mm in diameter with a volume of 125.6 mm^3^ were utilised for each individual mould. These were then subjected to an ethyl alcohol bath, to ensure complete disinfection, for a duration of 20 min. The three materials were prepared in accordance with the manufacturer’s instructions.

All the samples were then inserted into the previously prepared polyethylene moulds. A total of 18 samples were prepared and divided into 3 groups:

- Group 1 = consisting of 6 samples with GC Fuji IX^®^ (2 samples for each established time interval, *n* = 2).
- Group 2 = consisting of 6 samples with Biodentine^®^ (2 samples for each established time interval, *n* = 2).
- Group 3 = consisting of 6 samples with Biodentine XP 500^®^ (2 samples for each established time interval, *n* = 2).

In this study, a demineralizing solution was employed as an acidic medium reproducing the chemical aggressiveness of the oral environment, which under certain conditions may favour the development of carious lesions. The demineralising solution was composed of the following components: 0.2 M lactic acid, 3.0 mM CaCl_2_ and 1.8 mM KH_2_PO_4_ [15]. To ensure proper homogeneity, the solution was continuously stirred. Subsequent to the completion of the mixture, the pH was measured using a professional Hanna HI98190 pH meter (Darmstadt, Germany), resulting in a value of 4.5.

### 2.2. Measurement of Corrosion Rate

Following sample preparation, the initial weight of each specimen was recorded prior to its immersion in the demineralising solution. Two specimens from each material were preserved in their initial state as control samples. The residual specimens were meticulously transferred into containers containing 20 mL of demineralising solution (pH 4.5), following which they were stored within a temperature-controlled chamber set at 37 °C. Each of the three materials (GC FUJI IX^®^, Biodentine^®^ and Biodentine XP 500^®^) was subjected to testing on a total of four samples per material (a total of 12 samples). Of these, two samples per material were analysed at two time intervals (three days and seven days) for the corrosion rate and weight loss tests. The measurements were conducted with the aid of an analytical balance, utilised to ascertain the initial weight of the samples and their weights at the designated time intervals.

The weight loss method was employed to measure the corrosion rate of the three dental restorative materials. The method entails the quantification of the mass loss (in grams) of the material samples following their exposure to a corrosive environment with a pH of 4.5 at a temperature of 37 °C. This approach enables the direct observation of the materials’ reactivity under simulated oral cavity conditions. The fundamental principle of the method is predicated on the premise that a material undergoes mass loss as a consequence of chemical reactions that occur at the interface of the material with a corrosive medium (demineralizer). The accurate measurement of material mass is pivotal in determining the corrosion rate, and consequently, the clinical durability of the material. At predetermined time intervals (0 h, 3 days, 7 days), the weight of the samples was measured, as was the implicit mass loss. The samples were subjected to a drying process using filter paper, followed by a weighing procedure employing the AS 220/C/2 RADWAG (RADWAG Balances & Scales, Radom, Poland) analytical balance.

To determine the mass loss of the samples, the following formula was employed:

ΔW = Wi − Wf(1)

where

Wi = initial mass (recorded initially—0 h);

Wf = final mass (recorded at 3 days/7 days).

The corrosion rate was determined using the following formula [16]:

v = (K × ΔM)/(ρ × A × t)(2)

where

K = 8.76 × 10^4^; the constant used when expressing the corrosion rate v in mm/year;

ΔW = mass loss (g);

ρ = density of the material (g/cm^3^);

A = total surface area exposed (cm^2^);

T = exposure time expressed in hours (h).

### 2.3. Measurement of Vickers Hardness

**Figure 1:**
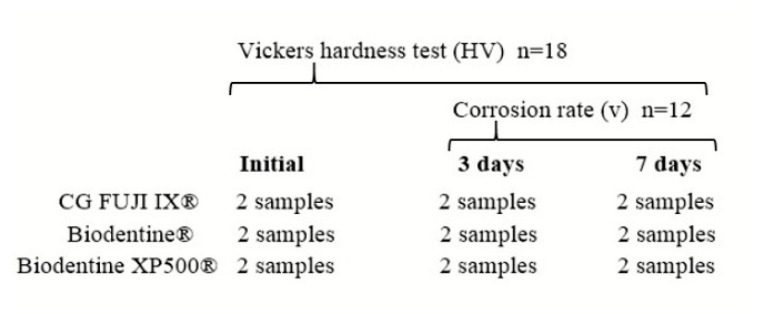
Schematic representation of the test and samples conducted in this study.

For the Vickers test, all six samples per material were tested (a total of 18 samples) (Figure 1). In order to facilitate the testing of the 18 samples, it was imperative that their upper and lower surfaces be maintained in a parallel orientation. The samples were prepared using polypropylene cylindrical moulds with dimensions of 13 mm length × 15 mm diameter. The items were subjected to a process of disinfection that involved their immersion in ethyl alcohol for a duration of 20 min.

The three materials were divided into groups and then placed into the previously prepared polypropylene moulds. In total, 18 samples were prepared in 18 moulds, divided into 3 groups.

- Group 1 = consisting of 6 samples (4 immersed and 2 non-immersed in acid solution) with GC Fuji IX^®^ material (2 samples for each established time interval, *n* = 2).
- Group 2 = consisting of 6 samples (4 immersed and 2 non-immersed in acid solution) with Biodentine^®^ material (2 samples for each established time interval, *n* = 2).
- Group 3 = consisting of 6 samples (4 immersed and 2 non-immersed in acid solution) with Biodentine XP 500^®^ material (2 samples for each established time interval, *n* = 2).

To embed the 18 samples, a self-polymerizing acrylic resin, Duracryl^TM^ (SpofaDental Inc., Jičín, Czechia) Plus was selected. After the curing process was complete, the Forcipol 202 (Metcon, Gaziantep, Turkey) device was used to remove excess resin material, ensuring that the surfaces of the newly obtained samples (upper and lower) were flat and parallel without altering the embedded sample.

The principle underlying the Vickers test is based on applying a fixed load to a pyramidal diamond indenter with an edge angle of 136°. In our study, a load of 300 g (0.3 kgf) was applied for 15 s. This ensured adequate pressure to create a visible and measurable imprint. After removing the indenter, a high-precision optical microscope was used to observe and measure the diagonals of the impressions (d1 and d2) remaining on the material surface. For each sample, 5 independent measurements were taken to calculate the average and minimise potential measurement errors. The major diagonal (d) of the indentation, in µm, was then used to calculate the Vickers hardness (HV). The formula for calculating the hardness is:

HV = F/S = (1.8544 × F)/d^2^, (3)

where

HV = Vickers hardness unit;

F = applied force in kilogram-force (kgf), 0.3 kgf;

S = surface area of the imprint;

d = the average diagonal measured in micrometres (µm).

To calculate the diagonal, the following formula was used:

d = (d1 + d2)/2 (4)

where

d_1_ = first diagonal of the indentation;

d_2_ = second diagonal of the indentation.

### 2.4. SEM Analysis

The SEM analysis was conducted utilising a VegaTescan LMH II (TESCAN, Brno-Kohoutovice, Czech Republic). The instrument was equipped with a secondary electron (SE) detector, a 2 kV heating gun voltage, a 15.5 mm working distance (WD), and a high vacuum setting. Two samples were selected from each material: one analysed in the initial state (immediately after setting) and the other after 7 days of immersion in the acid solution (in total 6 samples were examined). The samples were dried and then coated with gold to ensure the necessary conductivity for microscopy investigation. The gold coating (7 nm thick) was applied according to the standard protocol, ensuring a uniform coverage of the surface before placing the samples in the electron microscope.

### 2.5. Statistical Analysis

To determine whether there are statistically significant differences between the corrosion rates (v) and Vickers hardness (HV) of the three dental restorative materials at each interval, a one-way ANOVA analysis was conducted.

## 3. Results

**Figure 2:**
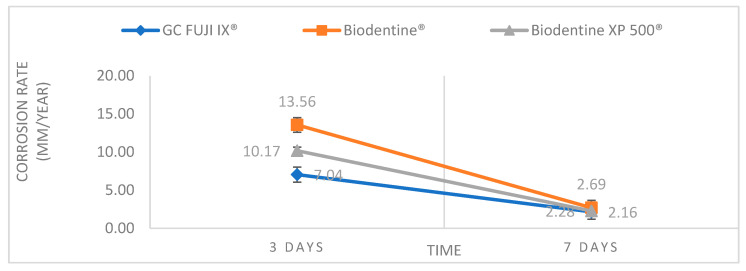
Corrosion rate of the three tested materials (GC Fuji IX^®^, Biodentine^®^, Biodentine XP 500^®^) after 3 and 7 days of immersion (error bars indicate the standard deviation).

This study investigated the chemical and mechanical stability of three dental materials by comparing their corrosion rates and Vickers hardness under simulated oral cavity conditions. The study aimed to provide information about their long-term behaviour and their ability to withstand the challenges of the oral environment. An essential aspect that justifies the need for enhanced corrosion resistance and mechanical strength, particularly for calcium silicate-based materials such as Biodentine^®^, lies in its clinical indications. According to current literature, Biodentine^®^ is indicated as a temporary restorative material in coronal cavities for up to six months and as a definitive restoration in root caries. These indications expose the material to continuous functional stress, acidic conditions and bacterial challenge—especially in high-risk patients. The results obtained indicated significant differences among the three materials regarding chemical stability. In general, Biodentine^®^ exhibited the highest corrosion rate, followed by Biodentine XP 500^®^, while GC FUJI IX^®^ demonstrated the best corrosion resistance (Figure 2). The high corrosion rate determined for Biodentine was found to be consistent with its elevated solubility, as previously reported by Kaup et al. (2015) [17]. Furthermore, the use of an acidic medium (pH = 4.5) in our study led to the acceleration of calcium-silicate dissolution, thereby explaining the higher mass loss and the resulting corrosion-rate value. The observations reported here are consistent with the findings of other studies in this field, which suggest that calcium silicate-based materials may be more susceptible to corrosion than glass-ionomer materials. This is attributed to the release of a greater number of ions from the former material, which is also related to a higher capacity for remineralisation. [18]. For instance, a study undertaken by Almutairi et al. in 2023 compared the solubility of pulp capping materials, concluding that calcium silicate-based materials exhibited higher solubility in distilled water and saline, suggesting a greater release of calcium ions [19]. Concurrently, a study undertaken by Somudorn et al. [15] in 2023 evaluated the remineralisation properties of calcium silicate- and glass-ionomer-based materials. The study concluded that Biodentine^®^ exhibited a superior remineralisation capacity in comparison to glass-ionomer cements (GC FUJI IX^®^) [20].

However, it is important to emphasise that the results of in vitro studies can be influenced by a number of factors, including the exact composition of the materials, the pH and composition of the demineralizing solution, temperature and duration of exposure.

**Figure 3:**
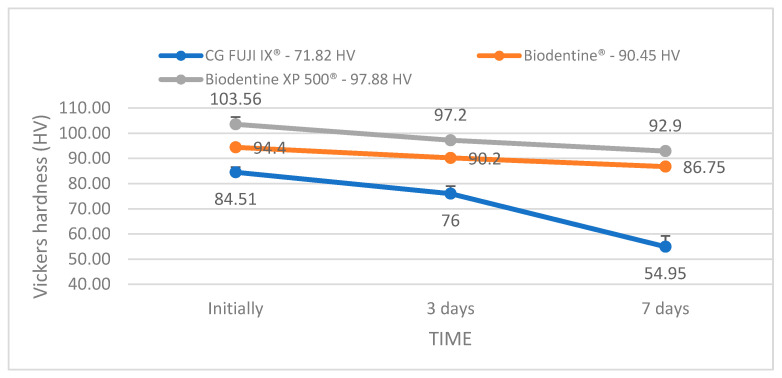
Variation in Vickers hardness of the three tested materials (GC Fuji IX^®^, Biodentine^®^, Biodentine XP 500^®^) after 3 and 7 days of immersion (error bars indicate the standard deviation).

**Figure 4:**
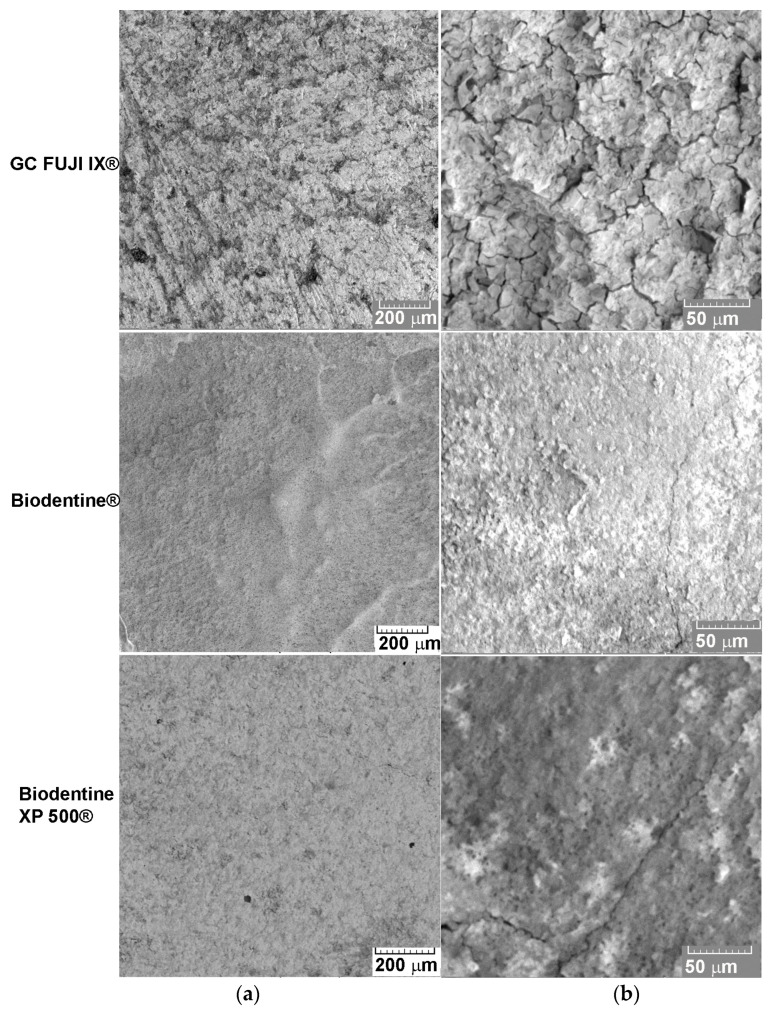
SEM images of GC FUJI IX^®^, Biodentine^®^ and Biodentine XP 500^®^ showing surface morphology: (**a**) initially at 100× magnification and (**b**) after 7 days at 500× magnification.

Regarding the Vickers hardness test, the ranking of the tested materials is slightly changed. The average values of the cumulative hardness over the three time intervals indicate the following order: Biodentine XP 500^®^ (97.88 HV), Biodentine^®^ (90.45 HV) and GC FUJI IX^®^ (71.82 HV) (Figure 3). Similarly, in the study by Kuru et al. (2024) Biodentine^®^ demonstrated superior chemical resistance compared to glass ionomers and even some composites, attributed to its composition and the hydration-based setting process involving calcium [21]. The resulting images (Figure 4) have provided relevant information about the microstructure of the tested materials and how they respond to the specific chemical stress of the acidic oral environment. In the case of the GC FUJI IX^®^ material, initial SEM images revealed a homogeneous surface with well-distributed grains and relatively compact topography. Following a period of seven days during which the sample was exposed to the acidic environment, discrete surface modifications became apparent. These included the formation of microcavities and slight disintegration of the matrix, but without any significant material loss. The findings of this study are in accordance with those of recent research, which has emphasised the conservative nature of glass ionomer cements in acidic environments, thus indicating a gradual and regulated deterioration [22].

As illustrated in Table 1, a series of comparative analyses were conducted among the groups using the post hoc Tukey test. This test was employed to assess the corrosion rate and Vickers hardness of GC FUJI IX^®^, Biodentine^®^, and Biodentine XP 500^®^ over the course of all designated time periods. 

Conversely, Biodentine XP 500^®^ exhibited a significantly more compact and uniform microstructure from the initial stage, with reduced porosity, in comparison to Bio-Dentine®. Following a period of seven days of exposure, the observed degradations were less pronounced, with localised modifications and an absence of structural collapse zones. The results of this in vitro study have significant clinical implications for the selection of dental restorative materials and for the approach to restorative treatment. Despite the fact that all of the materials that were examined demonstrated some degree of corrosion in the simulated acidic environment, the disparities observed between the materials suggest that certain materials may be more appropriate for particular clinical scenarios. One-way ANOVA analysis of corrosion rate and Vickers hardness for GC FUJI IX^®^, Biodentine^®^ and Biodentine XP 500^®^ at all the time periods was performed. 

The results obtained by One-way ANOVA test indicated significant statistical differences (*p* < 0.05) at certain time intervals: initially, 3 days, and 7 days.

In contrast, SEM images (Figure 4) of Biodentine^®^ showed more porous surface initially. After 7 days, profound surface modifications were observed, with large areas of erosion, microcracks, and detachment of fragments of material. The general appearance suggested a significant mass loss and severe alteration of the mineral matrix. 

**Figure 5:**
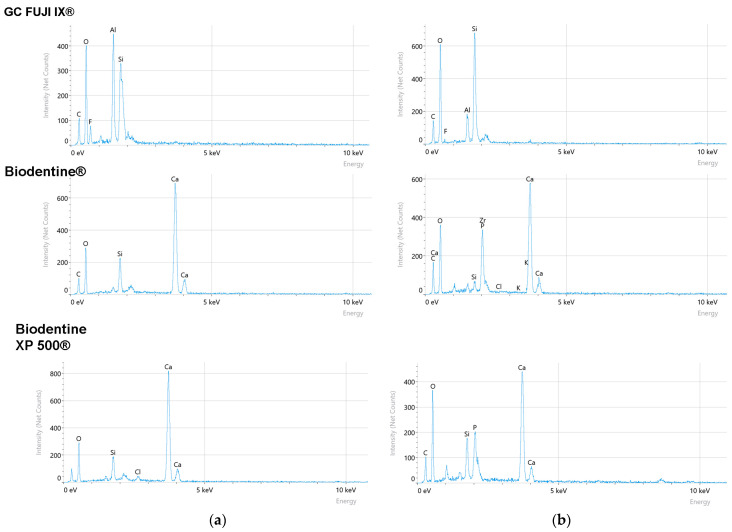
EDS Spectra of GC FUJI IX^®^, Biodentine^®^ and Biodentine XP 500^®^: (**a**) initially—T_0_ and (**b**) 7 days—T_2_.

To further characterise the materials, Energy-Dispersive X-ray Spectroscopy (EDS) analysis was conducted. The resulting elemental composition data are centralised and presented in Table 2 and EDS spectra in Figure 5.

## 4. Discussion

An important finding of this in vitro study is the observed difference between Bio-Dentine^®^ and Biodentine XP 500^®^. It has been established that both materials contain calcium silicate in their composition. However, it was also demonstrated that Biodentine XP 500^®^ exhibited a lower corrosion rate than Biodentine^®^. It is hypothesised that the observed changes in the composition and structure of Biodentine XP 500^®^ are the cause of the improved mechanical properties. The aforementioned alterations can be attributed to three factors: firstly, the incorporation of nanoparticles, secondly, adjustments to the calcium-to-silicate ratio, and thirdly, the addition of substances that prevent degradation.

The mechanisms by which dental materials are subjected to corrosion are complex and may involve the dissolution of soluble components, hydrolysis of chemical bonds and acid attack of the material matrix [23].

In the case of glass ionomers, the release of fluoride has been demonstrated to contribute to the remineralisation of dental structures and protection against carious lesions. However, under certain conditions, this process can also accelerate material degradation [24]. In the case of calcium silicate-based materials, the release of calcium ions can stimulate the formation of reparative dentin and promote healing [25]. However, this process may also result in rapid mass loss [26].

The clinical implications of the Vickers tests are significant. The chemical stability of dental restorative materials is an important factor in determining the longevity and success of restorations [27]. Materials with greater corrosion resistance are more likely to maintain marginal integrity, withstand mechanical loads and prevent bacterial infiltration and the development of carious lesions [28]. Therefore, the selection of appropriate restorative material should be based on a careful assessment of risks and benefits, considering the individual needs of the patient and the specific clinical situation.

The results of the SEM analysis are consistent with those pertaining to the corrosion rate, thereby corroborating the hypothesis that Biodentine^®^, despite its excellent biocompatibility, is more prone to degradation in an acidic environment. This behaviour has also been documented in other recent studies, which indicate increased solubility and intensive calcium ion release as part of the therapeutic mechanism [29]. However, the potential to compromise structural integrity in a hostile environment must be considered.

The compositional analysis via EDS of Biodentine XP 500^®^, Biodentine^®^ and GC FUJI IX^®^ samples revealed significant variations in the elemental composition of the materials’ surfaces after seven days (T_2_), compared to the initial state (T_0_). These modifications provide valuable insights into the degradation mechanism and interaction of the materials with the simulated oral environment.

A notable increase in the concentration of oxygen (O) was observed concurrently with a significant decrease in calcium (Ca) in the case of Biodentine XP 500^®^. This finding suggests the presence of a surface alteration, potentially resulting from hydration processes and the subsequent release of calcium ions into the surrounding medium. The reduced calcium level is indicative of material dissolution, a finding that is consistent with recent studies emphasising the degradation susceptibility of calcium silicate-based materials in aqueous environments [30]. The increased oxygen content may be attributable to the formation of calcium hydroxide or calcium carbonate on the surface, a consequence of interactions with the environment [31].

For Biodentine^®^, the EDS results indicated a marked decrease in silicon (Si) and a reduction in the level of calcium (Ca). This combination of silicon and calcium loss serves as an indicator of structural degradation within the material. As demonstrated in previous research, Bio-Dentine^®^ has been shown to release a significant quantity of calcium ions, thereby contributing to its bioactivity. This process has also been found to result in mass loss and increased porosity [32]. The degradation process of calcium silicate is a complex one, influenced by the pH and composition of its surrounding environment [33].

It was found that GC FUJI IX^®^ exhibited a significant increase in oxygen (O) and silicon (Si) content. This may be indicative of the formation of a silica-rich hydrated layer on the surface, which could offer a degree of protection against further degradation [2].

The EDS findings can be related to the corrosion and Vickers hardness testing results in order to provide a more comprehensive understanding of the material behaviours. The calcium and silicon loss observed in Biodentine^®^ and Biodentine XP 500^®^ is indicative of higher corrosion rates, suggesting a direct relationship between the elemental composition and material stability. The enhanced silicon content observed in GC FUJI IX^®^ may provide a rationale for its superior corrosion resistance.

The findings of the study demonstrated that GC FUJI IX® exhibited superior chemical stability in comparison to Biodentine® and Biodentine XP 500®, suggesting a slower and more controlled release of ions. In patients exhibiting elevated caries risk, such as those with exposed root surfaces or hard-to-reach interproximal areas, glass ionomers may represent a superior option due to their capacity to release fluoride and stimulate remineralisation [34]. In the context of restorations in deep cavities or in proximity to the pulp chamber, calcium silicate-based materials are often favoured due to their biocompatibility and ability to stimulate the formation of tertiary dentin [35]. Nevertheless, the findings demonstrated that Biodentine XP 500® displayed enhanced chemical stability in comparison with Biodentine®, thereby indicating that alterations to the composition of the material may potentially augment its corrosion resistance. 

The findings of this study lend support to the hypothesis that calcium silicate cement is more effective than glass ionomer cement in preserving surface hardness in an acidic environment. Biodentine XP 500® has been shown to exhibit consistent performance, thus establishing itself as an advanced restorative material with considerable clinical potential in challenging situations within the oral cavity. Notwithstanding the conclusive nature of the results, it is imperative to acknowledge that this study was conducted in a vitro setting, characterised by controlled conditions. In the clinical in vivo environment, the performance of materials can be significantly influenced by a number of factors. These include saliva, enzymes, pH variations, thermal cycling and occlusal forces. The findings of this study underline the significance of achieving an equilibrium between ion release and mechanical strength in the selection of dental restorative materials. The release of ions, specifically calcium silicates, facilitates tissue regeneration processes and contributes to the remineralisation of dental structures.

This research is preliminary because it has some limitations. The sample size can be considered small, which increases the influence of variation in the results. Though measurements were taken at multiple immersion intervals, the effect of time on the corrosion rate and microhardness may vary statistically. In addition, the ASTM corrosion method applied here was originally designed for metals and may not fully reflect the degradation behaviour of dental cements in acidic environments. 

Future studies should also investigate properties that are directly relevant to cement performance—such as abrasion resistance, water solubility, and water sorption—tested under similar acidic conditions to obtain a more complete insight on how these materials are degrading.

## 5. Conclusions

In conclusion, the three materials which were tested all exhibited favourable properties. The selection process was guided by the specific clinical requirements of the patient. GC FUJI IX^®^ exhibited superior chemical stability in comparison to Biodentine^®^ and Biodentine XP 500^®^. The investigation revealed that Biodentine XP 500^®^ exhibited enhanced mechanical stability in comparison to both Biodentine^®^ and GC FUJI IX^®^. Achieving an equilibrium between chemical stability and long-term mechanical strength is imperative. The selection of the most suitable material must be made after a thorough evaluation of the potential risks and benefits, with consideration given to the individual patient’s requirements and the particular characteristics of the clinical scenario (salivary pH, salivary flow, oral microbiota, dietary habits, masticatory forces, etc.).

## Figures and Tables

**Table 1:** Multiple comparisons between groups using the post hoc Tukey test for the corrosion rate and Vickers hardness for GC FUJI IX^®^, Biodentine^®^ and Biodentine XP 500^®^ at all the time periods.

	Time Periods	Comparison	Difference Between Means	MSE	SE	Q Value	HSD	Significant Difference
Corrosion rate	T_1_	Group 1 vs. Group 2	5.90	0.0000146	0.0022	8.60	0.01234	YES
		Group 1 vs. Group 3	12.47	0.0000153	0.0023	4.30	0.0118	YES
		Group 2 vs. Group 3	5.57	0.0000149	0.0024	1.89	0.0142	NO
	T_2_	Group 1 vs. Group 2	0.493	0.0000482	0.0021	2.60	0.0118	NO
		Group 1 vs. Group 3	0.246	0.0000096	0.0019	2.54	0.0166	NO
		Group 2 vs. Group 3	0.739	0.0000118	0.0011	0.0023	0.0142	NO
Vickers hardness	T_0_	Group 1 vs. Group 2	9.89	4.775	2.185	2.071	10.43	NO
		Group 1 vs. Group 3	19.05	65.06	8.066	2.362	524.66	NO
		Group 2 vs. Group 3	9.16	61.515	7.843	1.168	482.42	NO
	T_1_	Group 1 vs. Group 2	14.20	6.225	2.495	5.692	15.53	NO
		Group 1 vs. Group 3	21.20	6.155	2.481	8.546	15.26	YES
		Group 2 vs. Group 3	7.00	0.68	0.825	8.485	0.56	YES
	T_2_	Group 1 vs. Group 2	31.80	17.665	4.191	7.590	74.03	NO
		Group 1 vs. Group 3	37.95	17.745	4.212	9.009	74.74	NO
		Group 2 vs. Group 3	6.15	0.63	0.794	7.745	0.50	YES
T_0_ = initial; T_1_ = 3 days; T_2_ = 7 days Group 1 = GC Fuji IX^®^; Group 2 = Biodentine^®^; Group 3 = Biodentine XP 500^®^ MSE = Combined mean square error for the two compared groups SE = Standard error using MSE HSD = Honest Significant Difference (qxSE)								

**Table 2:** Elemental composition analysis using EDS for GC FUJI IX^®^, Biodentine^®^ and Biodentine XP 500^®^ at all the time periods.

Element	Wt. %					
	Biodentine XP 500^®^		Biodentine^®^		GC FUJI IX^®^	
	T_0_	T_2_	T_0_	T_2_	T_0_	T_2_
O	47.2	52.7	48.0	49.6	41.2	48.78
Si	5.7	5.2	6.3	1.3	14.1	23.2
Cl	1.4					
Ca	45.7	28.6	40.2	30.3		
C		7.8	5.5	10.0	17.1	17.9
P		5.7		8.0		
Al				0.8	15.3	5.1
F					12.3	5.02

## Data Availability

The original contributions presented in this study are included in the article. Further inquiries can be directed to the corresponding author.
